# A Systematic Review and Meta-Analysis of 35,409 Patients Undergoing PCI *versus* CABG for Unprotected Left Main Coronary Artery Diseases

**DOI:** 10.31083/j.rcm2508282

**Published:** 2024-08-09

**Authors:** Hao Liu, Dongdong Li, Chuncheng Gao, Huimiao Dai, Lin Kang, Mingming Zhang, Chen Yun, Wangang Guo

**Affiliations:** ^1^Department of Cardiology, The Second Affiliated Hospital of Air Force Medical University, 710038 Xi’an, Shaanxi, China; ^2^Department of Gastroenterology, The Second Affiliated Hospital of Air Force Medical University, 710038 Xi’an, Shaanxi, China; ^3^Department of Anesthesiology, The Second Affiliated Hospital of Air Force Medical University, 710038 Xi’an, Shaanxi, China

**Keywords:** UPLM, MACCEs, myocardial infarction, TVR

## Abstract

**Background:**

Patients with unprotected left main (UPLM) disease who underwent percutaneous coronary intervention (PCI) were found to have inconsistent results compared to those treated with coronary artery bypass grafting (CABG).

**Methods:**

We identified and enrolled randomized controlled trials (RCTs) and observational studies (OSs) comparing PCI *versus* CABG for UPLM disease. A meta-analysis was performed using Stata 17.0. The primary endpoints were major adverse cardiovascular and cerebrovascular events (MACCEs). Additionally, all-cause death, cardiac death, myocardial infarction (MI), stroke, target vessel revascularization (TVR), and stent thrombosis (ST) were included as secondary endpoints. The odds ratios and 95% confidence intervals (CIs) were calculated. Sensitivity analyses were implemented if $I^{2}$ > 50% or *p* < 0.01. Publication bias analysis was conducted if more than 10 studies were included.

**Results:**

A total of 5 RCTs and 18 OSs involving 35,409 patients were included. The CABG strategy had a significantly lower incidence of MACCEs, primarily due to TVR. A significantly lower stroke rate was observed with the PCI strategy, as well as a significantly lower all-cause death, cardiac death, MI, and ST rate compared with the CABG strategy.

**Conclusions:**

MACCE rates were significantly lower in patients who underwent CABG, primarily due to TVR, but stroke rates were higher. RCTs with different study types need further investigation to confirm the most effective strategy.

## 1. Introduction

The left main coronary artery (LMCA) supplies approximately 75 percent of the myocardium in the left ventricle. Left main lesions tend to have worse long-term outcomes and higher death rates than non-left main lesions. Coronary artery bypass grafting (CABG) has long been considered the best treatment strategy for LMCA disease. With the development of stent technology, the assistance of intraoperative imaging, the optimization of anti-thrombotic strategy, and the application of new drugs, there is an increasing use of percutaneous coronary intervention (PCI), with it gradually becoming an alternative treatment option [1]. In the American College of Cardiology (ACC)/American Heart Association (AHA)/Society for Cardiovascular Angiography and Interventions (SCAI) jointly published guidelines for coronary artery revascularization in 2021, CABG is recommended over PCI when revascularization is required for complex left main coronary artery disease [2]. Several randomized controlled trials (RCTs) have been performed to determine the optimum revascularization strategy for LMCA patients. Several studies have, however, found conflicting results when analyzing long-term results [3, 4, 5, 6, 7, 8]. According to the SYNTAX trial, PCI and CABG have similar 10-year all-cause death rates; CABG provides a significant survival advantage for three-vessel disease patients but not for left main disease [6]. The LE-MAIN trial demonstrated a positive long-term outcome for stenting in patients with left main lesions with moderate to low complex coronary artery disease [7]. The MAIN-COMPARE 10-year study found that CABG is superior in terms of death rate and a composite of death, myocardial infarction (MI), or stroke [8]. Therefore, these results are conflicting.

We reviewed the literature to determine which revascularization strategy, PCI or CABG, was superior for treating patients with left main disease. This trial was registered on https://inplasy.com/ (INPLASY202390009).

## 2. Methods

### 2.1 Literature Search

We comprehensively searched PubMed, Embase, Cochrane Database, and Web of Science and analyzed RCTs and observational studies (OSs) that compared PCI *versus* CABG in patients with unprotected left main (UPLM) disease between the initiation of the database and June 30, 2023. The key search terms included “left main”, “percutaneous coronary intervention”, and “coronary artery bypass graft”. The terms were searched for using the free combination method. All the obtained references were evaluated and screened by reading abstracts or full texts, and the eligible references were included. The search strategy is “left main and percutaneous coronary intervention and coronary artery bypass graft”. Details are shown in **Supplementary Table 1**.

### 2.2 Literature Inclusion and Exclusion Criteria

Inclusion criteria were (1) RCTs and OSs comparing PCI and CABG for left main bifurcation lesions; (2) studies that contained comparable data about the two treatment strategies; (3) at least one major adverse cardiovascular and cerebrovascular event (MACCE), all-cause death, cardiac death, MI, stroke, target vessel revascularization (TVR), and stent thrombosis (ST) as study outcomes; (4) a follow-up period of at least three years.

Exclusion criteria were (1) endpoint events of interest not being clearly reported or could not be extracted and calculated and (2) studies based on the same patient cohort.

### 2.3 Data Extraction

We independently reviewed the literature and extracted relevant data from two reviewers in our research group (HL and DL). CG (third reviewer) assisted in resolving any disagreements. Data extracted from the enrolled studies include (1) basic information, including first author, year of publication, data source, follow-up time, and research type; (2) participants’ characteristics, including the size of the sample, the mean age, and the gender ratio; (3) the extent of left main disease according to whether the left anterior descending (LAD)/left circumflex (LCX)/right coronary artery (RCA) were involved, only the LMCA, LMCA+single vessel disease (SVD), LMCA+double vessel diseases (DVD), LMCA+third vessel diseases (TVD); (4) outcomes including MACCEs, all-cause death, cardiac death, MI, stroke, TVR, and ST; (5) other information such as EuroSCORE, and SYNTAX scores.

### 2.4 Outcomes and Definitions

MACCEs, a composite of death, MI, stroke, and TVR, were the primary endpoint of this meta-analysis. Studies enrolled in the review varied in composition, and the initial definition was adopted in this review. **Supplementary Table 2** summarizes the definitions of all primary endpoints in each study. Components of ST and primary endpoint are secondary endpoints. These included all-cause death, cardiac death, MI, TVR, and stroke.

### 2.5 Risk Assessment of Bias

HL and DL conducted bias risk assessments. Any disparity was resolved through discussion with CG. The Cochrane Collaboration tool [9] was used to assess RCTs, while the Newcastle–Ottawa Quality Assessment Scale (NOS) [10] was used to assess OSs.

### 2.6 Statistical Analysis

Calculated odds ratios (OR) were based on 95% confidence intervals (CIs) using STATA/SE 17.0 (StataCorp LLC, College Station, TX, America). $I^{2}$ tests were used to examine heterogeneity between studies. Fixed-effects models were used in cases where *p* > 0.01 and $I^{2}$ < 50% rather than random-effects models. An analysis of heterogeneity and sensitivity was conducted to identify its origins. In the case of more than 10 studies, a regression-based Egger test was applied, and a non-parametric pruning and filling analysis was performed. *p*-values less than 0.05 were considered statistically significant.

## 3. Results

### 3.1 Baseline Data and Search Results

**Fig. 1.**
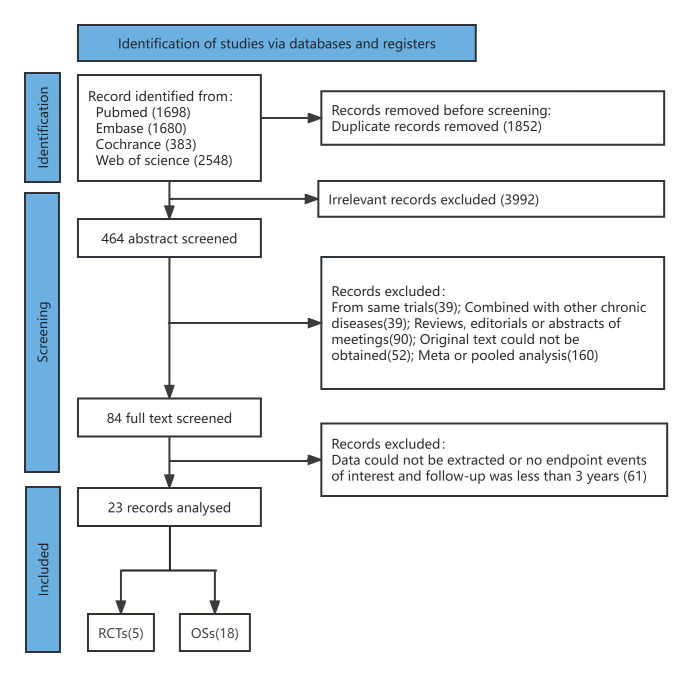
**Literature retrieval process**. RCTs, randomized control trials; OSs, observational studies.

As shown in Fig. 1, four databases were searched to identify the enrolled studies. From the 6350 searched studies, 1852 were excluded for being duplicates and 3992 for being irrelevant records. In the 464 screened abstracts, 30 records were from the same trials. A total of 39 records were combined with other chronic diseases; 90 records belonged to reviews/editorials/meeting abstracts, the original text could not be obtained in 52 records, and 160 were meta- or pooled analyses. In the 84 screened papers with full texts, the data could not be extracted in 61, or they had no referred endpoint events and no follow-up over 3 years. Finally, 23 studies were enrolled. Among these studies, 18 had MACCEs data, 21 had data on all-cause death, 11 had data on cardiac death, 20 had data on MI, 21 had data on stroke, 21 had data on TVR, and 5 had data on ST. The included studies reported research results between 1995 and 2015, and the publication dates ranged from 2010 to 2023. In total, 35,409 patients were enrolled in our study. Table 1 (Ref. [3, 4, 5, 6, 7, 11, 12, 13, 14, 15, 16, 17, 18, 19, 20, 21, 22, 23, 24, 25, 26, 27, 28]) lists the general characteristics of the studies. Table 2 (Ref. [3, 4, 5, 6, 7, 11, 12, 13, 14, 15, 16, 17, 18, 19, 20, 21, 22, 23, 24, 25, 26, 27, 28]) provides detailed patient and procedure information.

**Table 1.** **General characteristics of the enrolled studies**.

Study	Country/Territory	Center	Data from	Study period	Follow-up period	Study type
Park, *et al*. 2020 [4]	Korea	13 centers	PRECOMBAT trial	Apr.2004–Aug.2009	1,2,5,10 years	RCT
Buszman, *et al*. 2016 [7]	America	Multi-center	LE MANS trial	2001–2004	1,10 years	RCT
Thuijs, *et al*. 2019 [6]	18 North America and European countries	85 centers	SYNTAX trial	Mar.2005–Apr.2007	1,3,5,10 years	RCT
Holm, *et al*. 2020 [5]	9 northern European countries	36 centers	NOBLE trial	Dec.2009–Jan.2015	1,5 years	RCT
Stone, *et al*. 2019 [3]	17 countries	165 centers	EXCEL trial	Sep.2010–Mar.2014	3,5 years	RCT
Chieffo, *et al*. 2010 [11]	Milan	Single center	Local database	Mar.2002–Jul.2004	1,5 years	Non-RCT
Chieffo, *et al*. 2012 [12]	Milan	14 centers	The DELTA registry	Apr.2002–Apr.2006	1295 days	Non-RCT
Fukui, *et al*. 2014 [13]	Japan	Single center	Local database	Jan.2005–Dec.2010	37.1 months	Non-RCT
Guo, *et al*. 2017 [14]	China	Single center	Local database	Jan.2003–Jul.2009	7.1 years	Non-RCT
Huckaby, *et al*. 2022 [15]	America	Multi-center	Local database	2010–2018	5 years	Non-RCT
Jang, *et al*. 2022 [16]	Korea	Single center	Local database	Aug.2005–Apr.2013	7 years	Non-RCT
Jeong, *et al*. 2013 [17]	Korea	Single center	Local database	Jan.2001–Dec.2009	55.9 months	Non-RCT
Joy, *et al*. 2020 [18]	United Kingdom	Single center	Local database	Jan.2012–Dec.2012	5 years	Non-RCT
Lee, *et al*. 2017 [20]	9 Asian territories	50 centers	IRIS-MAIN registry	Jan.1995–Dec.2013	5.2 years	Non-RCT
Kang, *et al*. 2010 [19]	Korea	2 centers	Local database	Jan.2003–Dec.2006	0.5,1,2,3 years	Non-RCT
Lu, *et al*. 2016 [21]	China	Single center	Local database	Jan.2004–Dec.2010	4.3 years	Non-RCT
Persson, *et al*. 2023 [22]	Sweden	28 centers	SWEDENHEART registry	Jan.2005–Dec.2015	4.7 years	Non-RCT
Shiomi, *et al*. 2015 [23]	Japan	26 centers	The CREDO-Kyoto PCI/CABG registry cohort-2	Jan.2005–Dec.2007	3 years	Non-RCT
Wu, *et al*. 2010 [24]	China	Single center	Local database	Feb.2003–Dec.2006	4 years	Non-RCT
Yamamoto, *et al*. 2021 [25]	Japan	22 centers	The CREDO-Kyoto PCI/CABG registry cohort-3	Jan.2011–Dec.2013	5.5 years	Non-RCT
Yi, *et al*. 2012 [26]	Korea	Single center	Local database	Jul.2003–Jun.2007	38 months	Non-RCT
Yu, *et al*. 2020 [27]	China	Single center	Local database	Jan.2003–Jul.2009	12 years	Non-RCT
Zheng, *et al*. 2016 [28]	China	Single center	Local database	Jan.2004–Dec.2010	3 years	Non-RCT

RCT, randomized control trial; PRECOMBAT trial, Bypass Surgery Versus Angioplasty Using SirolimusEluting Stent in Patients With Left Main Coronary Artery Disease study; LE MANS trial, left main stenting trial; SYNTAX trial, Synergy Between PCI With Taxus and Cardiac Surgery trial; NOBLE trial, the Nordic-Baltic-British left main revascularisation study; EXCEL trial, Evaluation of XIENCE versus Coronary Artery Bypass Surgery for Effectiveness of Left Main Revascularization (EXCEL) trial; IRIS-MAIN registry, Interventional Research Incorporation Society-Left MAIN Revascularization (IRIS-MAIN) study; SWEDENHEART registry, Swedish Web-system for Enhancement and Development of Evidence-based care in Heart disease Evaluated According to Recommended Therapies; CREDO-Kyoto PCI/CABG registy, the Coronary Revascularization Demonstrating Outcome Study in Kyoto (CREDO-Kyoto) PCI/CABG; The DELTA registry, A Multicenter Registry Evaluating Percutaneous CoronaryIntervention Versus Coronary Artery Bypass Grafting for Left Main Treatment.

**Table 2.** **Baseline information of the enrolled patients and procedure**.

Study	Sample size	Age, year	Male, %	DM, %	Hypertension, %	Dyslipidemia, %	Current smoker, %	Prior PCI, %	Prior MI, %
Park, *et al*. 2020 [4]	300/300	61.8/62.7	76/77	34/30	54.3/51.3	42.3/40.0	29.7/27.7	12.7/12.7	4.3/6.7
Buszman, *et al*. 2016 [7]	52/53	60.0/61.3	60/73	19/17	75/70	65/60	-	-	37/32
Thuijs, *et al*. 2019 [6]	903/897	65.2/65.0	76/79	26/25	69/64	79/77	18/22	-	32/34
Holm, *et al*. 2020 [5]	592/592	66.2/66.2	80/76	15/15	65/66	-	19/22	20/20	-
Stone, *et al*. 2019 [3]	948/957	66.0/65.9	76.2/77.5	30.2/28.0	74.5/73.9	71.5/69.3	24.1/20.8	18.4/15.9	18.1/16.9
Chieffo, *et al*. 2010 [11]	107/142	63.6/67.5	-	18.7/23.2	58.8/76.0	70.0/69.0	49.5/59.1	-	-
Chieffo, *et al*. 2012 [12]	1874/900	65.8/66.5	73.9/63.6	27.7/34.0	64.0/67.7	61.8/64.7	45.2/42.7	24.8/13.7	-
Fukui, *et al*. 2014 [13]	29/409	69.4/69.1	79.3/76.8	41.4/37.7	69.0/64.3	75.9/61.6	27.6/55.0	-	20.7/35.7
Guo, *et al*. 2017 [14]	149/110	61.9/60.7	74.5/74.5	28.2/29.1	55.7/52.7	48.3/39.1	47.7/45.5	-	18.1/25.5
Huckaby, *et al*. 2022 [15]	134/215	74/74	64.9/64.6	41.0/41.9	88.1/88.0	82.8/88.4	17.2/16.0	35.1/35.0	46.3/47.4
Jang, *et al*. 2022 [16]	118/112	64.0/64.3	75/69	31/39	56/63	38/24	25/20	12/14	3/5
Jeong, *et al*. 2013 [17]	346/553	62.5/62.5	78.3/75.2	28.3/37.1	44.8/60.0	18.5/37.1	24.0/30.0	25.4/17.0	14.5/12.1
Joy, *et al*. 2020 [18]	14/74	69/71	92/71	12/14	36/29	-	14/0	-	-
Lee, *et al*. 2017 [20]	2850/2337	62.7/63.5	74.7/76.1	32.4/37.6	57.1/57.2	42.1/38.9	25.2/28.5	17.2/12.2	8.1/13.5
Kang, *et al*. 2010 [19]	205/257	64.2/65.7	70.2/73.9	37.6/43.6	63.4/67.3	54.6/59.5	43.4/49.4	22.4/3.5	4.4/3.1
Persson, *et al*. 2023 [22]	1773/9364	72.8/69.6	71.8/79.0	19.1/20.9	64.3/63.8	52.9/61.2	13.3/15.6	7.5/5.2	19.1/16.3
Lu, *et al*. 2016 [21]	208/270	70/69	84.1/85.6	47/46	78/83	54/50	50/67	-	-
Shiomi, *et al*. 2015 [23]	365/640	71.4/69.4	71/77	42/45	86/85	-	21/25	-	19/16
Wu, *et al*. 2010 [24]	131/245	61.9/63.6	76/83	27/29	65/62	32/31	8/11	-	12/15
Yamamoto, *et al*. 2021 [25]	383/472	72.3/70.2	75.2/79.7	42.6/48.7	82.5/81.4	-	17.8/14.0	-	19.8/18.6
Yi, *et al*. 2012 [26]	243/269	62.3/65.0	72/77	32.9/38.6	57.2/57.2	-	-	-	-
Yu, *et al*. 2020 [27]	271/201	61.7/60.6	74.2/76.1	28.8/28.9	56.1/50.2	49.8/38.8	48.3/46.2	14.0/11.9	17.3/26.9
Zheng, *et al*. 2016 [28]	1442/2604	59.9/62.2	78.6/82.0	24.1/31.0	54.2/64.3	50.1/59.1	46.5/53.6	22.5/9.7	23.6/38.1

DM, diabetes mellitus; PCI, percutaneous coronary intervention; MI, myocardial infarction.

**Table 2.** **Continued**.

LVEF, %	EuroSCORE (PCI)	EuroSCORE (CABG)	SYNTAX score (PCI)	SYNTAX score (CABG)	LMCA only, %	LMCA+SVD, %	LMCA+DVD, %	LMCA+TVD, %	RCA involvement
61.7 ± 8.3/60.6 ± 8.5	2.6 ± 1.8	2.8 ± 1.9	24.3 ± 9.6	25.3 ± 10.9	9.0/11.3	16.7/17.7	33.7/30.0	40.7/41.0	-
53.5 ± 10.7/53.7 ± 6.7	3.3 ± 8.7	3.5 ± 2.3	25.2 ± 8.7	24.7 ± 6.8	56/60	13/6	27/19	60/75	-
EF <30%, 1/3	3.8 ± 2.6	3.8 ± 2.7	28.4 ± 11.5	29.1 ± 11.4	12/14	19/20	31/30	38/35	-
60/60	median, 2	median, 2	22.5 ± 7.5	22.4 ± 8.0	81/81	-	-	-	-
57.0 ± 9.6/57.3 ± 9.0	-	-	20.6 ± 6.2	20.5 ± 6.1	-	-	-	-	-
52.0 ± 10.4/52.2 ± 11.4	4.4 ± 3.6	4.3 ± 3.4	28.8 ± 10.4	29.4 ± 5.78	-	-	-	-	40.4/69.0
53.8 ± 12/53.3 ± 11.5	4.9 ± 3.6	5.1 ± 2.6	-	-	-	64.6/84.2	-	-	36.5/69.7
56.3 ± 10.1/57.1 ± 11.2	8.8 ± 10.9	7.6 ± 10.4	-	-	20.7/2.9	44.8/8.1	13.8/24.9	20.7/64.1	-
64/62	5 (3–6)	5 (3–6)	-	-	18.1/6.4	26.8/15.5	30.2/27.3	24.8/50.9	-
55/53	-	-	-	-	-	-	-	-	-
-	1.4 ± 1.4	1.9 ± 1.2	20.7 ± 8.2	33.1 ± 10.4	15/3	27/9	31/38	26/51	-
EF <40, 8.4/12.8	-	-	24 ± 9	28 ± 9	-	-	-	-	-
52 ± 13/42 ± 10	-	-	24 ± 9	25 ± 8	-	-	-	-	-
59.7 ± 9.8/56.7 ± 11.3	-	-	-	-	16.2/4.5	24.4/8.9	32.4/22.4	27.0/64.1	-
55.5 ± 11.6/55.4 ± 12.5	4.2 ± 3.9	5.6 ± 3.8	-	-	14.6/5.8	31.7/8.9	20.5/24.9	33.2/60.3	54.6/73.9
-	-	-	-	-	10.3/4.4	28.2/11.1	33.2/26.7	28.3/57.8	-
49 ± 12/49 ± 12	7.1 ± 5.1	6.4 ± 4.0	-	-	4.3/1.9	21/4.8	31/18	44/75	56/85
EF <40%, 12/9.5	-	-	26.5 (21–34)	30 (22–40)	8.5/8.9	24.5/16.9	36.0/28.4	31.0/45.8	-
60.0 ± 11.8/59.2 ± 12.0	4.2 ± 2.7	4.3 ± 2.4	-	-	9/3	18/4	38/26	35/67	44/82
59.4 ± 14.1/61.6 ± 13.0	-	-	27.5 (22–36)	31 (23–38)	6.5/0	24.8/1.3	33.2/26.9	35.5/71.8	-
EF <40, 11.9/12.6	-	-	-	-	-	-	-	-	-
64/62	5 (3–6)	5 (3–6)	-	-	19.9/7.0	27.3/15.9	30.3/25.9	22.5/51.2	-
63.1 ± 7.2/60.2 ± 8.2	1.8 ± 1.8	2.8 ± 2.1	23.6 ± 6.7	33.3 ± 7.8	7.4/0.7	21.1/4.7	37.1/15.8	34.5/78.7	47.1/86.4

LVEF, left ventricular ejection fractions; EF, ejection fractions; LMCA, left main coronary artery; SVD, single vessel disease; DVD, double vessel disease; TVD, third vessel disease; RCA, right coronary artery; PCI, percutaneous coronary intervention; CABG, coronary artery bypass grafting.

### 3.2 Study Quality Assessment

**Fig. 2.**
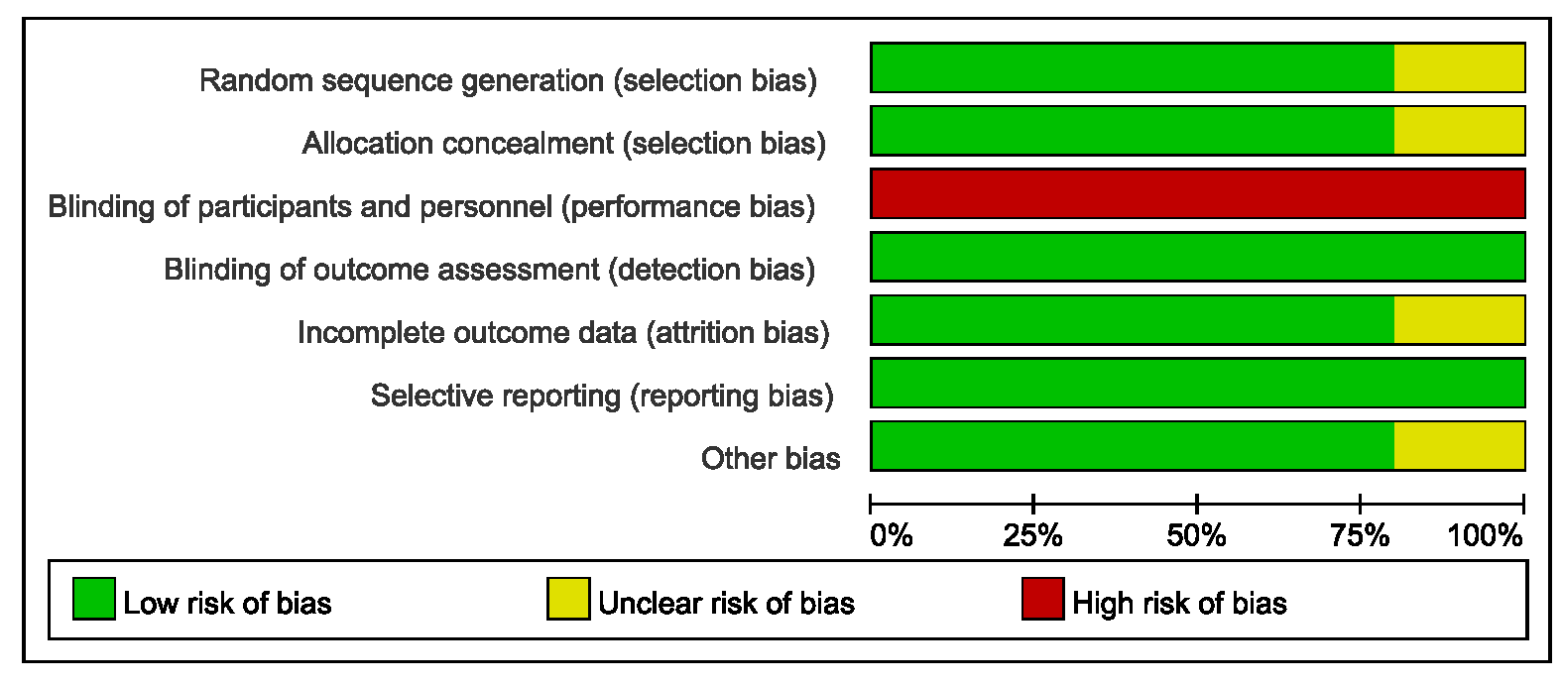
**RCTs quality assessment using the Cochrane Collaboration tool. **RCTs, randomized control trials.

The Cochrane Collaboration tool was used to evaluate the quality of the RCTs. Fig. 2 shows the seven criteria for the five RCTs. In Table 3 (Ref. [11, 12, 13, 14, 15, 16, 17, 18, 19, 20, 21, 22, 23, 24, 25, 26, 27, 28]), NOS judged all 18 observational studies to be low-risk.

**Table 3.** **OSs quality assessment using the NOS**.

Study	Selection	Comparability	Outcome	Total score
Chieffo, *et al*. 2010 [11]	★★★	★★	★★★	8
Chieffo, *et al*. 2012 [12]	★★★	★★	★★★	8
Fukui, *et al*. 2014 [13]	★★★	★★	★★	7
Guo, *et al*. 2017 [14]	★★★	★★	★★	7
Huckaby, *et al*. 2022 [15]	★★★	★★	★★	7
Jang, *et al*. 2022 [16]	★★★	★★	★★	7
Jeong, *et al*. 2013 [17]	★★★	★★	★★★	8
Joy, *et al*. 2020 [18]	★★★★	★★	★★★	9
Lee, *et al*. 2017 [20]	★★★	★★	★★★	8
Kang, *et al*. 2010 [19]	★★★	★★	★★★	8
Lu, *et al*. 2016 [21]	★★★	★★	★★	7
Persson, *et al*. 2023 [22]	★★★	★★	★★	7
Shiomi, *et al*. 2015 [23]	★★★	★★	★★★	8
Wu, *et al*. 2010 [24]	★★★	★★	★★★	8
Yamamoto, *et al*. 2021 [25]	★★★	★★	★★	7
Yi, *et al*. 2012 [26]	★★★	★★	★★	7
Yu, *et al*. 2020 [27]	★★★	★★	★★	7
Zheng, *et al*. 2016 [28]	★★★	★★	★★★	8

NOS, Newcastle–Ottawa Quality Assessment Scale; OSs, observation studies.

### 3.3 Primary Endpoint

#### Major Adverse Cardiac and Cerebrovascular Events

**Fig. 3.**
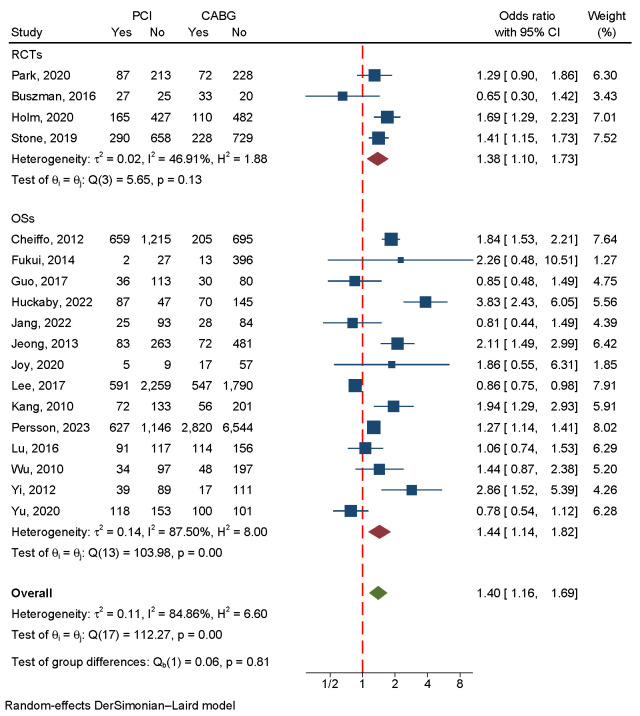
**An analysis of the forest plots of major adverse cardiac and cerebrovascular events between PCI and CABG strategies**. RCTs, randomized controlled trials; OSs, observational studies; PCI, percutaneous coronary intervention; CABG, coronary artery bypass grafting.

**Fig. 4.**
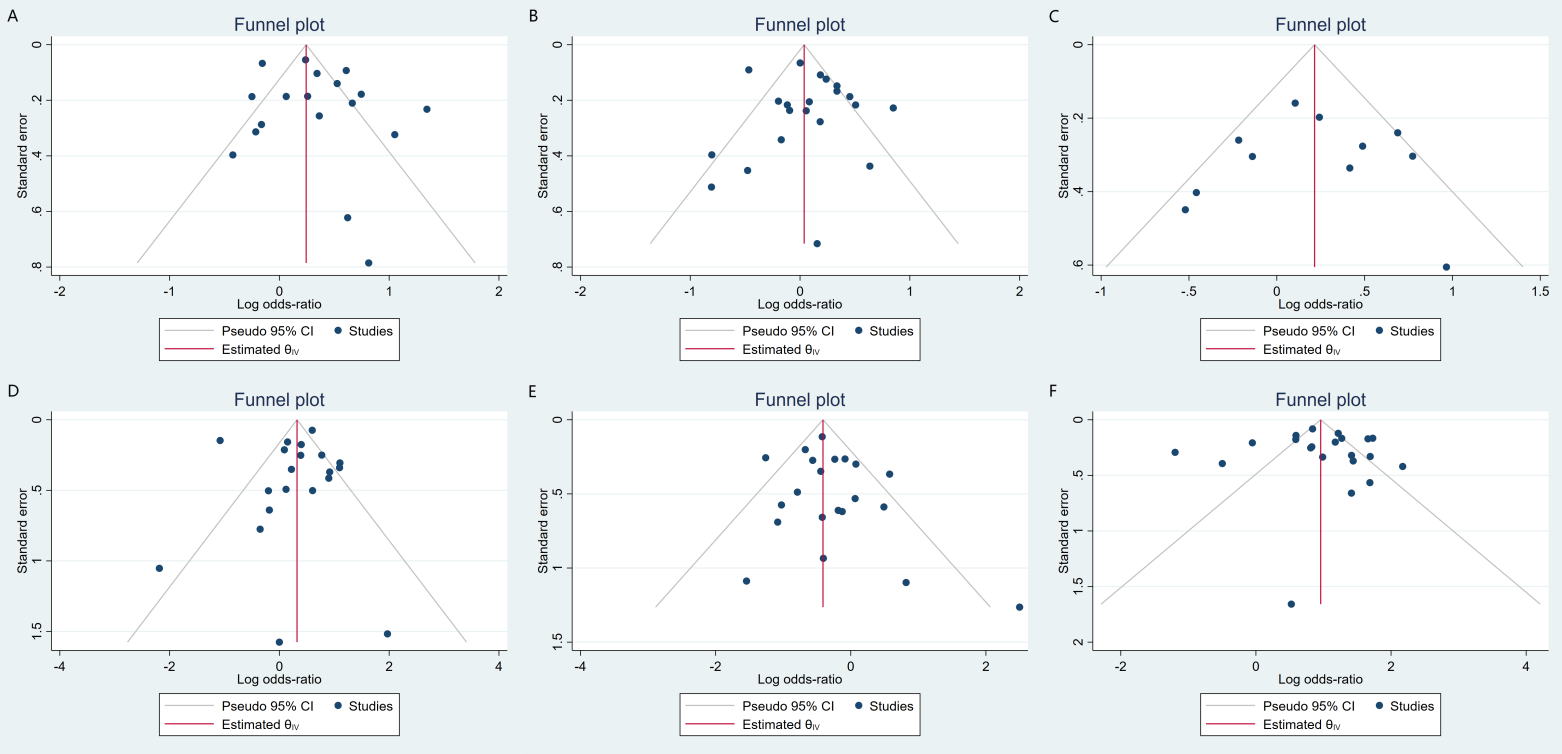
**Contour-enhanced funnel plots are used to evaluate the publication bias of studies about major adverse cardiac and cerebrovascular events (A), all-cause death (B), cardiac death (C), myocardial infarction (D), stroke (E), and stent thrombosis (F)**.

**Fig. 5.**
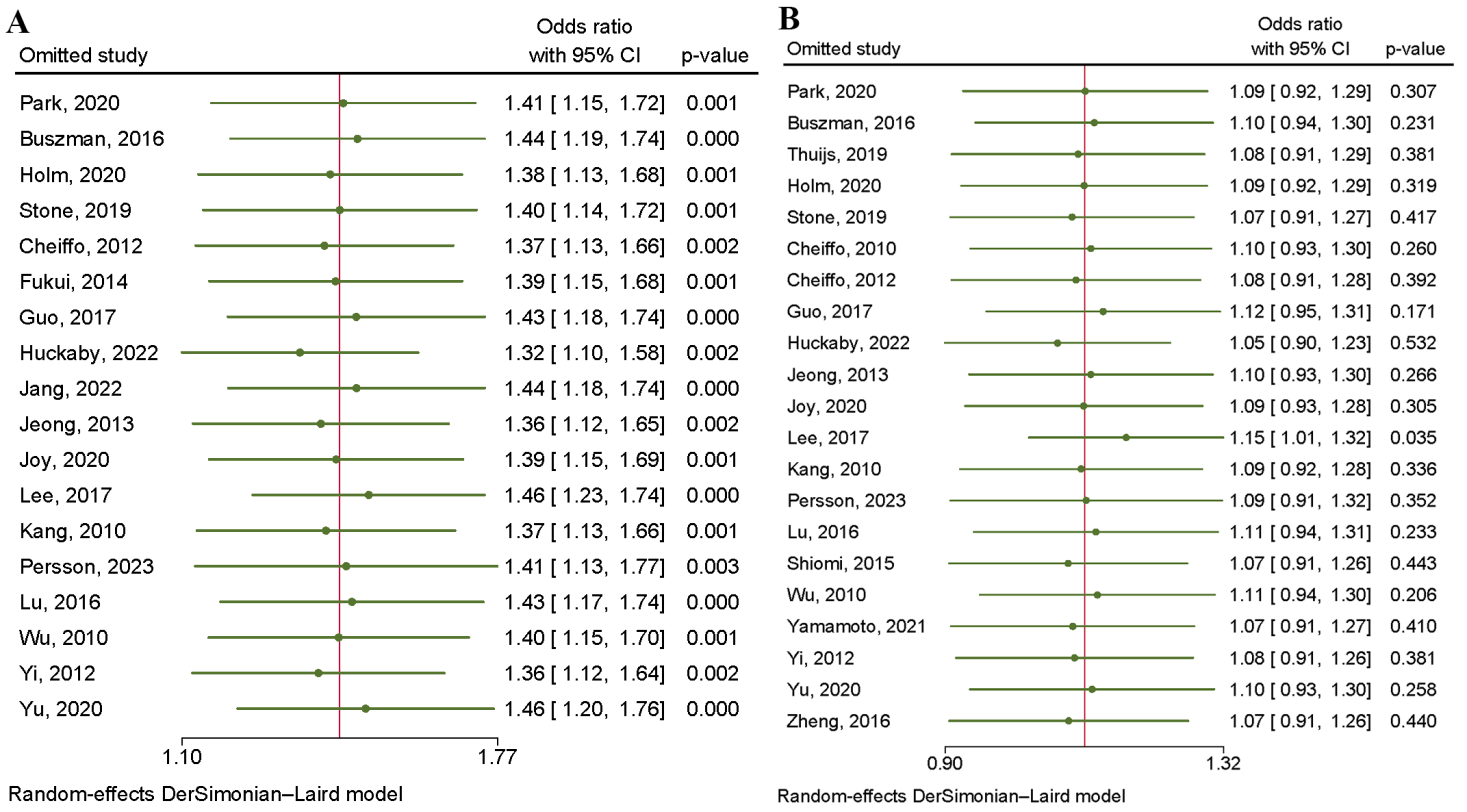
**Heterogeneity of studies sensitivity analysis about major adverse cardiac and cerebrovascular events (A) and all-cause death (B)**.

There was a significant difference between CABG and PCI in the four RCTs and 14 OSs that included 26,929 patients. Since there was a large amount of heterogeneity in the data ($I^{2}$ = 84.86%, *p* < 0.001), a random-effects model was used (Fig. 3). Neither a funnel plot nor a regression-based Egger test showed evidence of publication bias (*p* = 0.67) (Fig. 4A). According to the sensitivity analysis, heterogeneity was mainly caused by three studies [15, 20, 27] (Fig. 5A). Eliminating these three studies greatly reduced heterogeneity, and the subsequent results matched the primary results (**Supplementary Fig. 1**).

### 3.4 Secondary Endpoints

#### 3.4.1 All-Cause Death

**Fig. 6.**
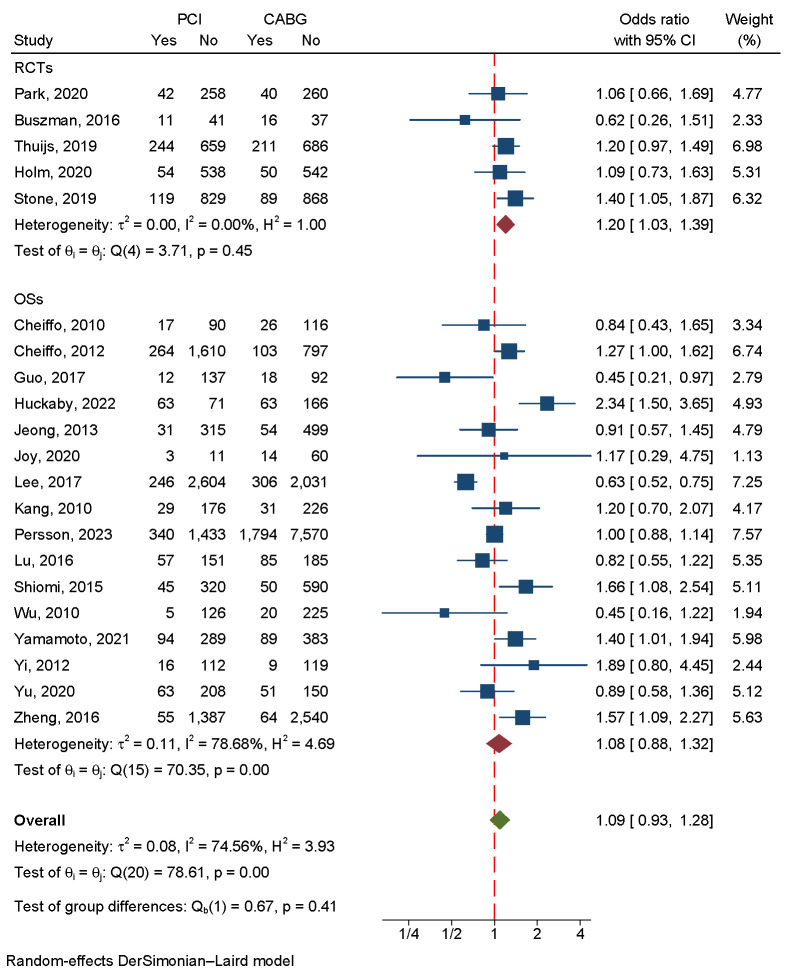
**An analysis of the forest plots of all-cause death between the PCI and CABG strategies**. RCTs, randomized controlled trials; OSs, observational studies; PCI, percutaneous coronary intervention; CABG, coronary artery bypass grafting.

A total of 35,505 patients were enrolled in five RCTs and 16 OSs. The results indicated that the CABG strategy was not statistically significant but associated with lower all-cause mortality. We used a random-effects model due to the large heterogeneity ($I^{2}$ = 74.56%, *p* < 0.001) (Fig. 6). Egger’s regression-based test and funnel plots showed no apparent publication bias (*p* = 0.40) (Fig. 4B). A sensitivity analysis revealed that heterogeneity was largely caused by three studies [15, 20, 23] (Fig. 5B). Eliminating these three studies greatly reduced heterogeneity, and the subsequent results matched the preceding results (**Supplementary Fig. 2**).

#### 3.4.2 Cardiac Death

**Fig. 7.**
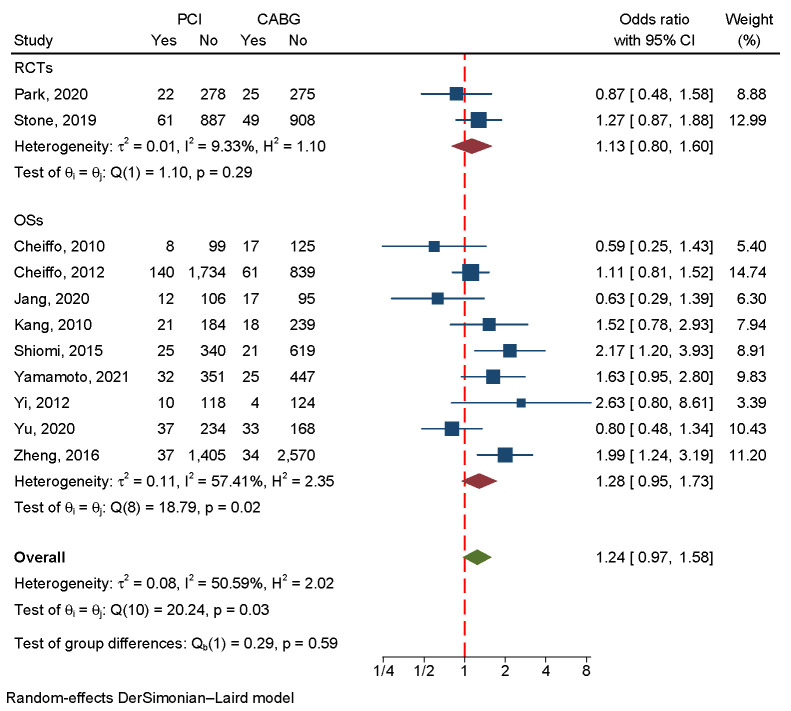
**An analysis of the forest plots of cardiac death between PCI and CABG strategies**. RCTs, randomized controlled trials; OSs, observational studies; PCI, percutaneous coronary intervention; CABG, coronary artery bypass grafting.

The incidence of cardiac death was assessed in two RCTs and nine OSs involving 12,854 patients. The results favored the CABG strategy for significantly lower cardiac death. Fixed-effect models were used because the heterogeneity was acceptable ($I^{2}$ = 50.59%, *p* = 0.03) (Fig. 7). A regression-based Egger test and funnel plots revealed no publication bias (*p* = 0.88) (Fig. 4C).

#### 3.4.3 Myocardial Infarction

**Fig. 8.**
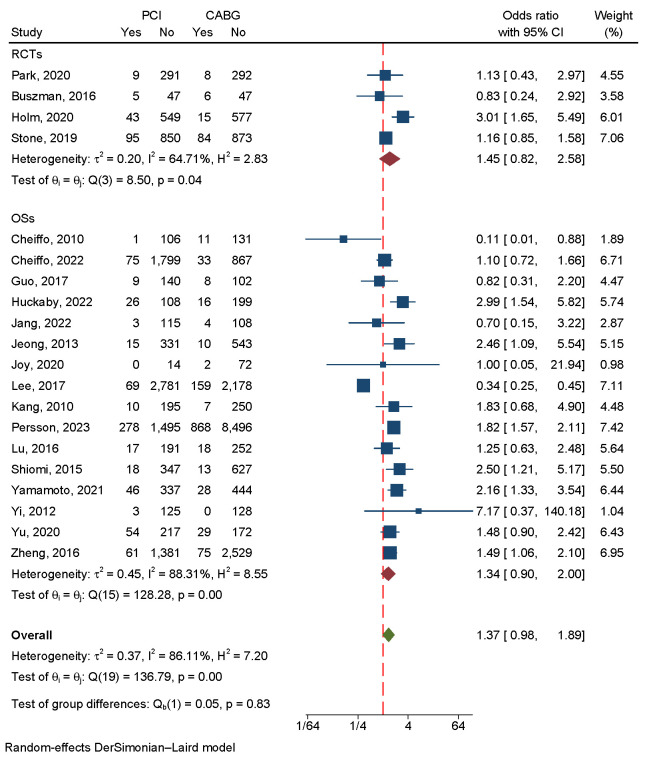
**An analysis of the forest plots of myocardial infarction between PCI and CABG strategies**. RCTs, randomized controlled trials; OSs, observational studies; PCI, percutaneous coronary intervention; CABG, coronary artery bypass grafting.

**Fig. 9.**
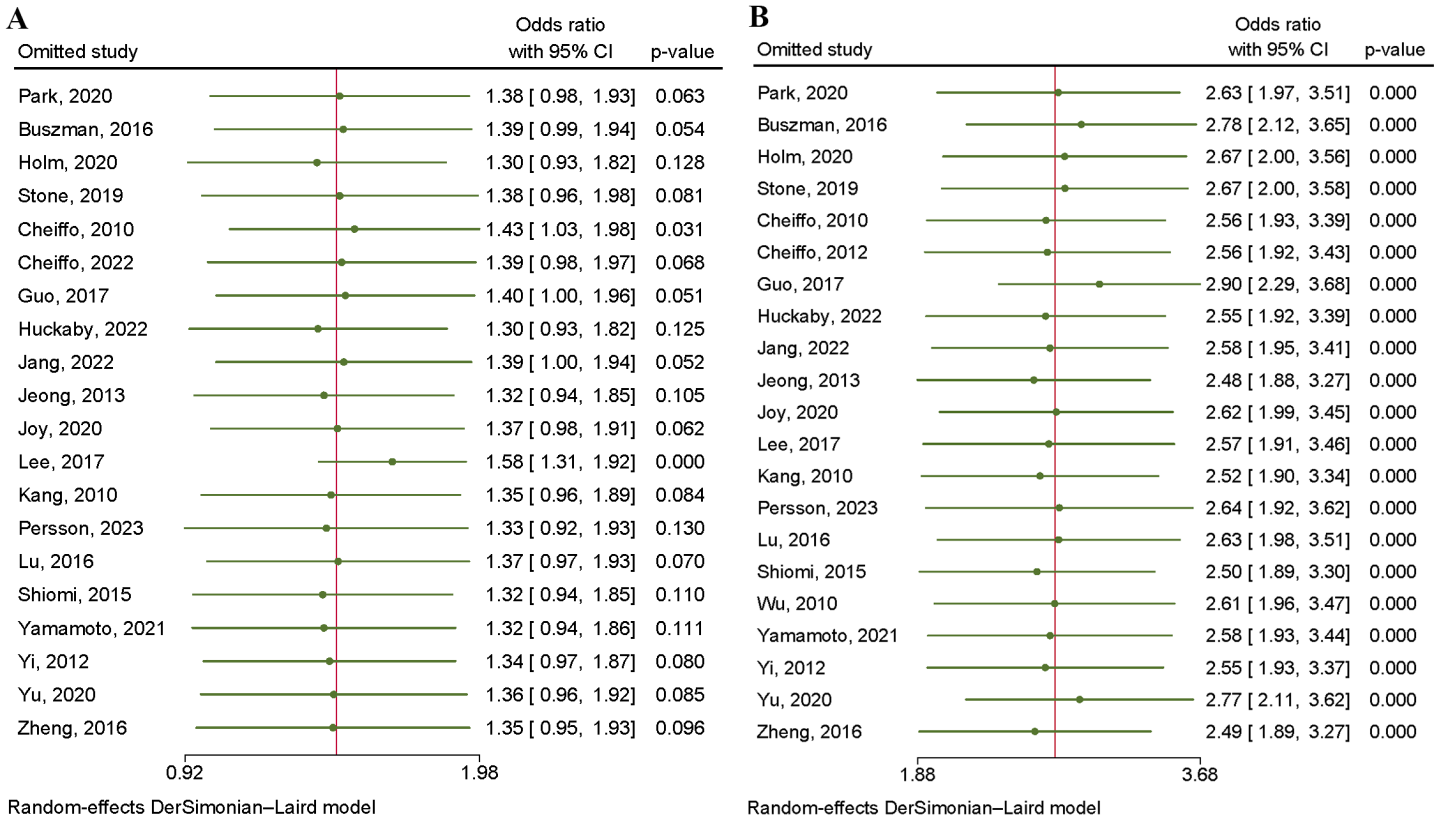
**Heterogeneity of studies sensitivity analysis about myocardial infarction (A) and target vessel revascularization (B)**.

A total of 32,540 patients were included in four RCTs and 16 OSs to evaluate the incidence of MI. The overall effect showed that the CABG strategy has a lower incidence of myocardial infarction. As a result of the heterogeneity, a random-effects model was used ($I^{2}$ = 86.11%, *p* < 0.001) (Fig. 8). A regression-based Egger test and funnel plots revealed no publication bias (*p* = 0.66) (Fig. 4D). A sensitivity analysis revealed that heterogeneity was largely caused by three studies [5, 15, 20] (Fig. 9A). Eliminating these three studies greatly reduced heterogeneity, and the subsequent results matched the preceding results (**Supplementary Fig. 3**).

#### 3.4.4 Stroke

**Fig. 10.**
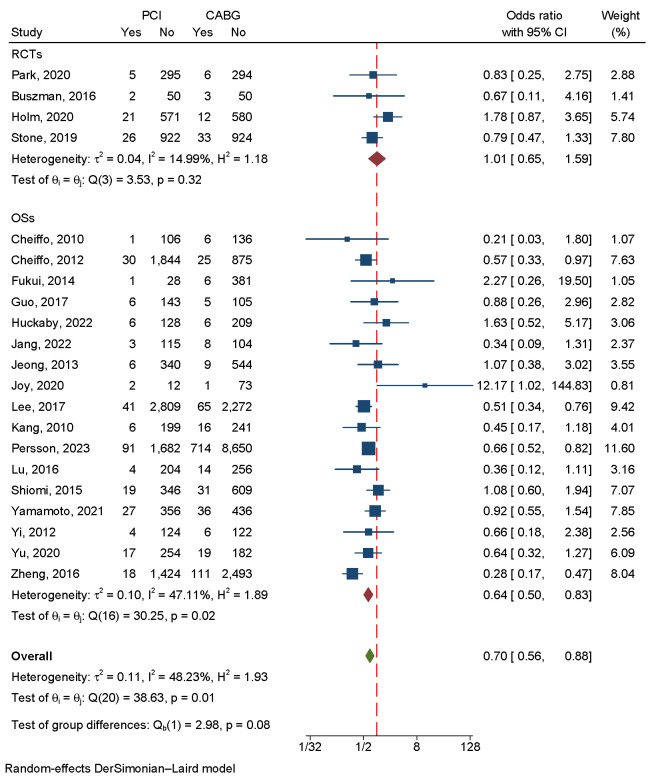
**An analysis of the forest plots of stroke between PCI and CABG strategies**. RCTs, randomized controlled trials; OSs, observational studies; PCI, percutaneous coronary intervention; CABG, coronary artery bypass grafting.

A total of 32,708 patients were included in four RCTs and 17 OSs investigating the incidence of stroke. The overall effect was found to be that the PCI strategy had a significantly lower incidence of stroke. Fixed-effects models were used because heterogeneity was relatively small ($I^{2}$ = 48.23%, *p* = 0.01) (Fig. 10). A regression-based Egger test and funnel plots revealed no publication bias (*p* = 0.25) (Fig. 4E).

#### 3.4.5 Target Vessel Revascularization

**Fig. 11.**
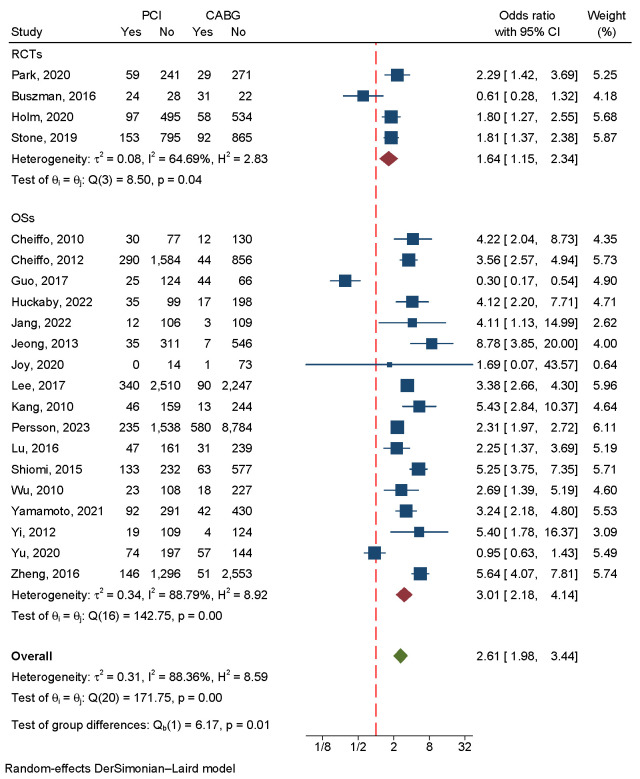
**An analysis of the forest plots of target vessel revascularization between PCI and CABG strategies**. RCTs, randomized controlled trials; OSs, observational studies; PCI, percutaneous coronary intervention; CABG, coronary artery bypass grafting.

A total of 32,646 patients were included in four RCTs and 17 OSs investigating the incidence of TVR. A significant advantage of CABG over PCI was found in the overall analysis. Since there was a large amount of heterogeneity in the data ($I^{2}$ = 88.36%, *p* = 0.00), we used a random-effects model (Fig. 11). A regression-based Egger test and funnel plots revealed no publication bias (*p* = 0.79) (Fig. 4F). A sensitivity analysis revealed that heterogeneity was largely caused by the studies of Buszman 2016, Guo 2017, Jeong 2013, Yu 2020 and Zheng 2016 [7, 14, 17, 27, 28] (Fig. 9B). Eliminating these five studies greatly reduced heterogeneity, and the subsequent results matched the previous results (**Supplementary Fig. 4**).

#### 3.4.6 Stent Thrombosis

**Fig. 12.**
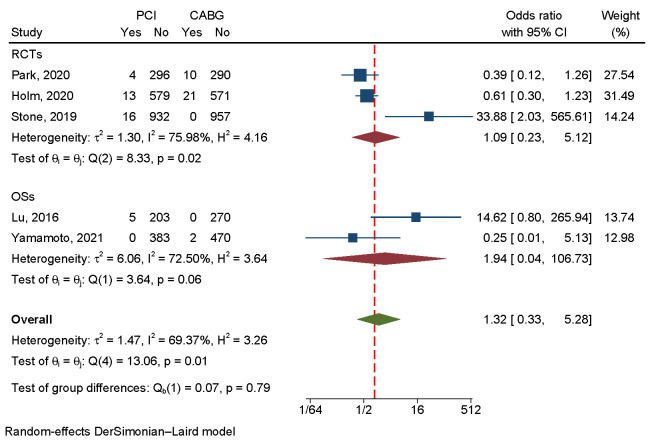
**An analysis of the forest plots of stent thrombosis between PCI and CABG strategies**. RCTs, randomized controlled trials; OSs, observational studies; PCI, percutaneous coronary intervention; CABG, coronary artery bypass grafting.

Three RCTs and two OSs involving 5022 patients reported the incidence of ST. PCI and CABG did not show a significant difference in ST (Fig. 12). Due to the small number of included studies, funnel plot and sensitivity analyses were not performed.

## 4. Discussion

This study included five RCTs and 18 OSs. For the primary endpoints of MACCEs, the PCI strategy was associated with a higher incidence of MACCEs, and there was a considerable degree of heterogeneity between strategies, which mainly occurred from the three OSs. The three OSs had conflicting conclusions regarding MACCEs, TVR, and all-cause death. We assumed that the heterogeneity of the three OSs may be closely related to their duration of follow-up. The study by Yu *et al.* (2020) [27] had a follow-up period of twelve years. Another two studies, Huckaby **et al*. *(2022) [15] and Lee* et al*. (2017) [20], were followed for only five years. PCI might have shown an advantage after a longer follow-up period. In addition, these three studies made appropriate statistical adjustments to compensate for the non-randomized design of the observational study. However, some other factors that might have resulted in differences in clinical outcomes were not considered.

In subgroup analyses, MACCEs, cardiac death, MI, stroke, TVR, and ST were all consistently associated with aggregated ORs in RCTs and OSs. We did not identify publication bias when analyzing primary and secondary endpoint events. As shown in Table 4, the aggregated OR values of all endpoints indicated that the CABG strategy was associated with significantly lower MACCE rates, which were primarily driven by TVR. The PCI strategy was associated with significantly lower stroke rates. There was also a lower tendency for the CABG strategy to result in all-cause death, cardiac death, MI, and ST, although the differences were not statistically significant. Based on these results and considering the importance of survival, CABG might be a better strategy.

**Table 4.** **Summarized OR values for all endpoints**.

		Aggregated OR (RCTs)	Aggregated OR (OSs)	Aggregated OR (Overall)
Primary endpoint				
MACCEs		1.38*	1.44*	1.40*
Secondary endpoints				
	All-cause death	1.20*	1.08	1.09
	Cardiac death	1.13	1.28	1.24
	MI	1.45	1.34	1.37
	Stroke	1.01	0.64*	0.70*
	TVR	1.64*	3.01*	2.61*
	ST	1.09	1.94	1.32

*, *p* < 0.05 (PCI strategy *vs*. CABG strategy). Yellow cell, favoring the PCI strategy. Blue cell, favoring the CABG strategy. MACCEs, major adverse cardiac and cerebrovascular events; TVR, target vessel revascularization; MI, myocardial infarction; ST, stent thrombosis; OR, odds ratio; RCTs, randomized controlled trials; OSs, observational studies; PCI, percutaneous coronary intervention; CABG, coronary artery bypass grafting.

In 2023, Feng *et al*. [29] published a systematic review comparing the two multi-vessel coronary artery (MVCA) or left main (LM) strategies, including nine studies involving 8621 patients. In that review, CABG improved five-year survival among patients with MVCA but not in those with LM. Consequently, we compared PCI and CABG for UPLM disease patients based on their long-term prognosis. A recent study by Persson *et al*. [22] in 2023 was added to our review to enlarge the sample size.

Benedetto *et al*. [30] analyzed Bayesian networks for LM disease. According to this network meta-analysis, DES did not result in better PCI outcomes than CABG. Moreover, the results of current trials did not clarify whether drug-eluting stents (DESs) are as safe as CABG in patients with cardiovascular events. Therefore, further research involving the use of the new generation DESs for LM diseases is warranted to help clinicians determine the optimal strategy for managing UPLM disease.

Although CABG had fewer overall MACCEs in treating LM lesions, this advantage was mainly driven by ST and TVR, not all-cause death. This might have been because the combined effects of CABG surgery, anesthesia, and cardiopulmonary bypass offset its advantage in coronary revascularization. Since there was an increase in mortality in patients undergoing CABG, PCI is often recommended as the therapy of choice. Drug-eluted balloon therapy may provide an advantage for PCI since it acts as a bridge to facilitate stent implantation in the stenotic vessel. At present, proprotein convertase subtilisin/kexin 9 (PCSK9) inhibitors and PCSK9 small interfering ribonucleic acid (siRNA) drugs have shown excellent results in preventing cardiovascular events in coronary heart disease. With the popularization of endovascular imaging technology, TVR may be further reduced when PCI is adopted. The widespread use of novel oral antiplatelet agents in LM may also further reduce the risk of ST. In conclusion, minimally invasive strategies have a significant role in treating LM lesions, although technical issues still need to be resolved.

## 5. Limitations

A major limitation in our review is the variable definition of endpoints across the studies. The study period only ranged from 1995 to 2015, and a few randomized controlled trials were included. The data extracted was only at the time of publication. SYNTAX scores and the EuroSCORE have been regarded as important predictors of cardiovascular survival; however, we could not better analyze the outcomes after PCI or CABG due to the limited data. Another weakness is that the limitations of the observational studies themselves cannot account for the preferences of physicians in selecting treatment strategies, which indirectly results in patients being given CABG or PCI. Finally, this review not have a prepared study protocol.

## 6. Conclusions

MACCE rates were significantly lower in patients who underwent CABG, primarily due to TVR, but stroke rates were higher. RCTs need to be further investigated to determine the most effective strategy.

## Data Availability

In the review, the template data collection forms, data extracted from the included studies, the data used for all analyses, as well as the analytical code are available to all.

## Abbreviations

PCI, percutaneous coronary intervention; CABG, coronary artery bypass grafting; UPLM, unprotected left main; RCTs, randomized controlled trials; OSs, observational studies; MACCEs, major adverse cardiac and cerebrovascular events; MI, myocardial infarction; TVR, target vessel revascularisation; ST, stent thrombosis; ACC, American College of Cardiology; AHA, American Heart Association; SCAI, Society for Cardiovascular Angiography and Interventions; LAD, left anterior descending; LCX, left circumflex; RCA, right coronary artery; LMCA, left main coronary artery; ESC, European Society of Cardiology; EACTS, European Association for Cardio-Thoracic Surgery; SVD, single vessel disease; DVD, double vessel disease; TVD, third vessel disease; NOS, Newcastle Ottawa Quality Assessment Scale; OR, odds ratio; CI, confidence interval; MVCA, multi-vessel coronary artery; DESs, drug-eluting stents; PCSK9, proprotein convertase subtilisin/kexin 9; siRNA, small interfering ribonucleic acid.

## Supplementary Material

Supplementary material associated with this article can be found, in the online version, at https://doi.org/10.31083/j.rcm2508282.
