# Supplementary material for: A Systematic Review and Meta-Analysis of 35,409 Patients Undergoing PCI versus CABG for Unprotected Left Main Coronary Artery Diseases
Source: Rev Cardiovasc Med. 2024 Aug 9;25(8):282. doi: 10.31083/j.rcm2508282 (PMC11367015; doi:10.31083/j.rcm2508282)
Supplement: Supplementary file 1 [file 2153-8174-25-8-282-s1.zip › 2153-8174-25-8-282-s1/Supplementary Materials.docx]

**Supplementary Table 1.** Literature searching strategy.

| Database | Searching strategy |
| --- | --- |
| PubMed | ("Left main or Left Main Coronary Artery Disease or Left Main Disease or Left Main Diseases or Left Main Coronary Disease"[Title/Abstract] and "Percutaneous Coronary Intervention[MeSH terms] or Percutaneous Coronary Intervention or Coronary Intervention, Percutaneous or Coronary Interventions, Percutaneous or Intervention, Percutaneous Coronary or Interventions, Percutaneous Coronary or Percutaneous Coronary Interventions or Percutaneous Coronary Revascularization or Coronary Revascularization, Percutaneous or Coronary Revascularizations, Percutaneous or Percutaneous Coronary Revascularizations or Revascularization, Percutaneous Coronary or Revascularizations, Percutaneous Coronary[Title/Abstract]"and "Coronary artery bypass[MeSH terms] or Coronary artery bypass or Artery Bypass, Coronary or Artery Bypasses, Coronary or Bypasses, Coronary Artery or Coronary Artery Bypasses or Coronary Artery Bypass Surgery or Bypass, Coronary Artery or Aortocoronary Bypass or Aortocoronary Bypasses or Bypass, Aortocoronary or Bypasses, Aortocoronary or Bypass Surgery, Coronary Artery or Coronary Artery Bypass Grafting[Title/Abstract]") |
| Embase | ("Left main or Left Main Coronary Artery Disease or Left Main Disease or Left Main Diseases or Left Main Coronary Disease"[Title/Abstract] and Percutaneous Coronary Intervention or Coronary Intervention, Percutaneous or Coronary Interventions, Percutaneous or Intervention, Percutaneous Coronary or Interventions, Percutaneous Coronary or Percutaneous Coronary Interventions or Percutaneous Coronary Revascularization or Coronary Revascularization, Percutaneous or Coronary Revascularizations, Percutaneous or Percutaneous Coronary Revascularizations or Revascularization, Percutaneous Coronary or Revascularizations, Percutaneous Coronary[Title/Abstract] and Coronary artery bypass or Artery Bypass, Coronary or Artery Bypasses, Coronary or Bypasses, Coronary Artery or Coronary Artery Bypasses or Coronary Artery Bypass Surgery or Bypass, Coronary Artery or Aortocoronary Bypass or Aortocoronary Bypasses or Bypass, Aortocoronary or Bypasses, Aortocoronary or Bypass Surgery, Coronary Artery or Coronary Artery Bypass Grafting[Title/Abstract]") |
| Cochrane Database | ("Left main or Left Main Coronary Artery Disease or Left Main Disease or Left Main Diseases or Left Main Coronary Disease"[Title/Abstract] and "Percutaneous Coronary Intervention[MeSH terms] or Percutaneous Coronary Intervention or Coronary Intervention, Percutaneous or Coronary Interventions, Percutaneous or Intervention, Percutaneous Coronary or Interventions, Percutaneous Coronary or Percutaneous Coronary Interventions or Percutaneous Coronary Revascularization or Coronary Revascularization, Percutaneous or Coronary Revascularizations, Percutaneous or Percutaneous Coronary Revascularizations or Revascularization, Percutaneous Coronary or Revascularizations, Percutaneous Coronary[Title/Abstract]"and "Coronary artery bypass[MeSH terms] or Coronary artery bypass or Artery Bypass, Coronary or Artery Bypasses, Coronary or Bypasses, Coronary Artery or Coronary Artery Bypasses or Coronary Artery Bypass Surgery or Bypass, Coronary Artery or Aortocoronary Bypass or Aortocoronary Bypasses or Bypass, Aortocoronary or Bypasses, Aortocoronary or Bypass Surgery, Coronary Artery or Coronary Artery Bypass Grafting[Title/Abstract]") |
| Web of science | ("Left main or Left Main Coronary Artery Disease or Left Main Disease or Left Main Diseases or Left Main Coronary Disease"[Title/Abstract] and Percutaneous Coronary Intervention or Coronary Intervention, Percutaneous or Coronary Interventions, Percutaneous or Intervention, Percutaneous Coronary or Interventions, Percutaneous Coronary or Percutaneous Coronary Interventions or Percutaneous Coronary Revascularization or Coronary Revascularization, Percutaneous or Coronary Revascularizations, Percutaneous or Percutaneous Coronary Revascularizations or Revascularization, Percutaneous Coronary or Revascularizations, Percutaneous Coronary[Title/Abstract] and Coronary artery bypass or Artery Bypass, Coronary or Artery Bypasses, Coronary or Bypasses, Coronary Artery or Coronary Artery Bypasses or Coronary Artery Bypass Surgery or Bypass, Coronary Artery or Aortocoronary Bypass or Aortocoronary Bypasses or Bypass, Aortocoronary or Bypasses, Aortocoronary or Bypass Surgery, Coronary Artery or Coronary Artery Bypass Grafting[Title/Abstract]") |

**Supplementary Table 2.** Definitions of MACCE in different studies.

| Study | Definitions |
| --- | --- |
| Park, 2020 | MACCE was defined as a composite of death from any cause, MI, stroke, or ischemia-driven target vessel revascularization. |
| Buszman, 2016 | MACCE included total mortality, myocardial infarction, target vessel revascularization, and stroke. |
| Thuijs, 2019 | MACCE includes all-cause death, cerebrovascular event (stroke), documented nonfatal MI, and revascularization by percutaneous intervention or bypass surgery. |
| Holm, 2019 | MACCE comprising all-cause mortality, non-procedural myocardial infarction, repeat revascularisation, or stroke. |
| Stone, 2019 | The primary composite end point of death from any cause, stroke, or myocardial infarction. |
| Cheiffo, 2010 | MACCE were analyzed : cardiac death, MI, cerebrovascular events, target lesion revascularization (TLR), and target vessel revascularization (TVR). |
| Cheiffo, 2012 | Major adverse cardiac and cerebrovascular event was defined as the composite endpoint of death, CVA, MI, and TVR. |
| Fukui, 2014 | MACCE was defined as death from any cause or stroke, myocardial infarction, or repeat revascularization. |
| Guo, 2017 | MACCE: the composite of cardiac death, MI, stroke or repeat revascularization. |
| Huckaby, 2021 | MACCE which included allcause mortality, stroke, myocardial infarction (MI), and repeat revascularization. |
| Jang, 2021 | MACE defined as a composite of cardiac death, spontaneous myocardial infarction (MI), stroke, and target vessel revascularization (TVR). |
| Jeong, 2013 | MACCE including death, myocardial infarction or stroke and target-vessel revascularization. |
| Joy, 2020 | MACE being a composite of stroke, myocardial infarction (MI), target vessel revascularisation and all-cause mortality. |
| Lee,2017 | MACCE was defined as a composite of all-cause death, myocardial infarction (MI),stroke, or repeat revascularization. |
| Kang, 2010 | Major adverse cardiac and cerebrovascular events were defined as a composite of death, myocardial infarction, cerebrovascular accident, or TVR. |
| Lu, 2016 | MACCE defined as all-cause death, MI, stroke,and clinically driven repeat revascularization. |
| Persson, 2023 | MACCE were defined as death, MI, stroke, or new revascularization (whichever occurred first) within the follow-up period. |
| Shiomi, 2015 | The primary outcome measure in the current study was a composite of death, MI, and stroke. |
| Wu, 2010 | MACCEs were defined as the occurrence of death, nonfatal myocardial infarction, TVR, or stroke. |
| Yamamoto, 2021 | The primary outcome measure of this study was all-cause death. |
| Yi, 2012 | MACCEs included all-cause mortality, stroke, nonfatal MI and target vessel revascularization (TVR). |
| Yu, 2020 | MACCE (major adverse cardiac and cerebrovascular events; the composite of cardiac death,non-procedural MI,stroke or repeat revascularization). |
| Zheng, 2016 | The primary outcome measure was 3-year all-cause mortality after the initial revascularization. |
| MI, myocardial infarction; TLR, target lesion revascularization; ST, stent thrombosis; TVR, target vessel revascularization; CVA, Cardiovascular accident. | |


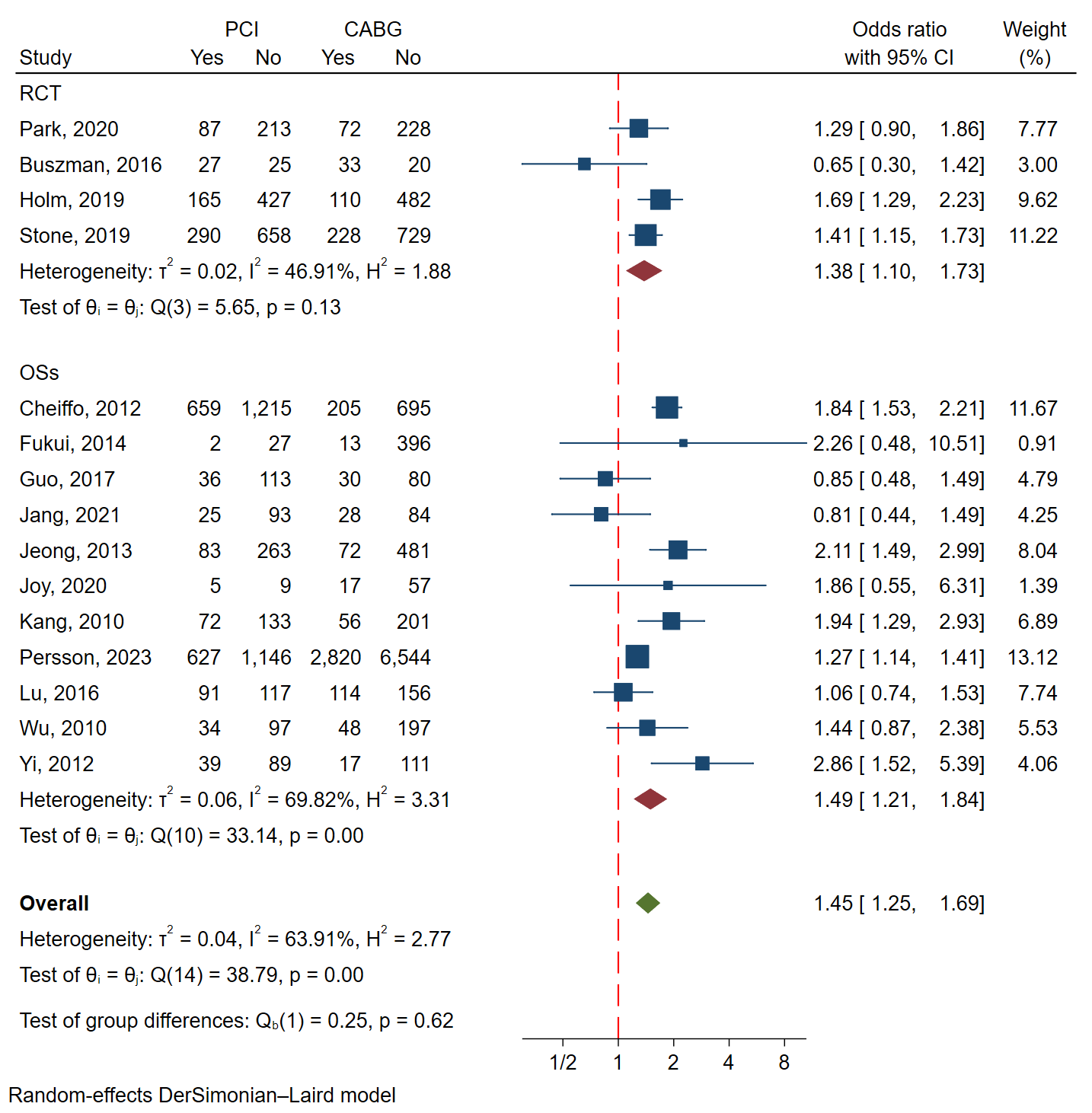


**Supplementary Fig. 1. An analysis of the forest plots of major adverse cardiac and cerebrovascular events between PCI strategy and CABG strategy.** RCT, randomized controlled trail; OSs, observational studies.


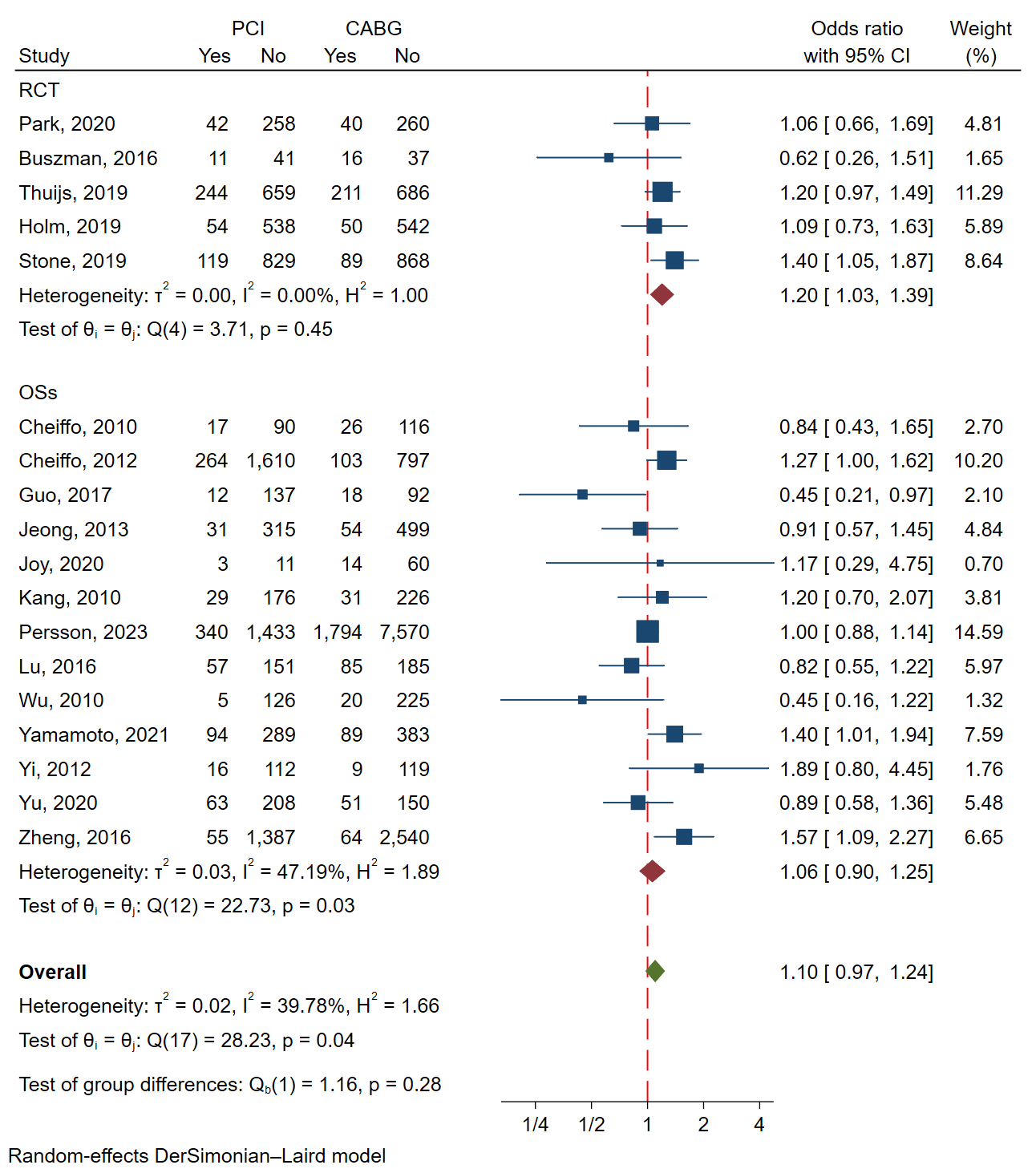


**Supplementary Fig. 2. An analysis of the forest plots of all-cause death between PCI strategy and CABG strategy.** RCT, randomized controlled trail; OSs, observational studies.


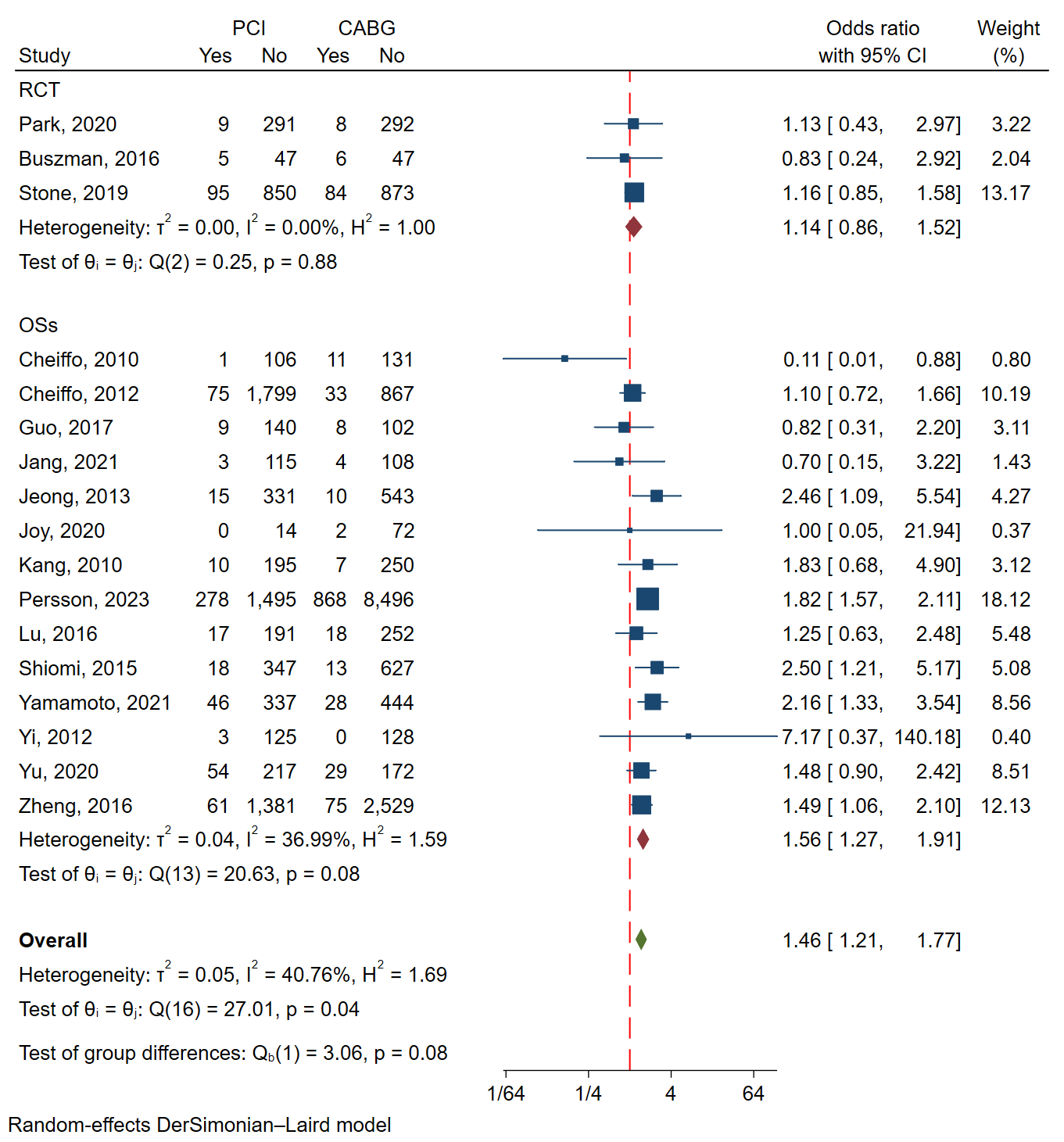


**Supplementary Fig. 3. An analysis of the forest plots of myocardial infarction between PCI strategy and CABG strategy.** RCT, randomized controlled trail; OSs, observational studies.


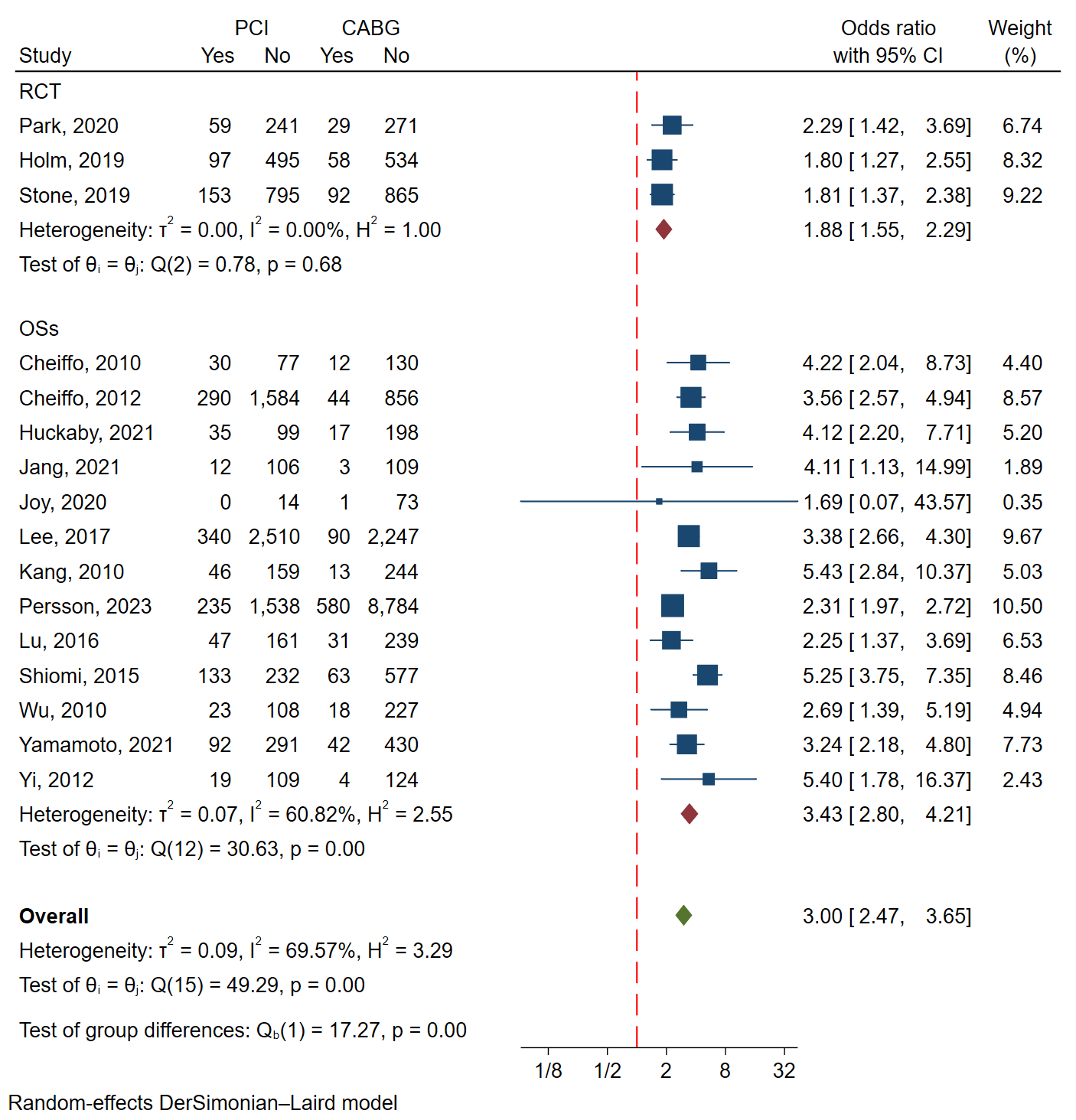


**Supplementary Fig. 4. An analysis of the forest plots of target vessel revascularization between PCI strategy and CABG strategy.** RCT, randomized controlled trail; OSs, observational studies.
